# Infectious Disease Management through Point-of-Care Personalized Medicine Molecular Diagnostic Technologies

**DOI:** 10.3390/jpm2020050

**Published:** 2012-05-02

**Authors:** Luc Bissonnette, Michel G. Bergeron

**Affiliations:** 1 Département de microbiologie-infectiologie et d'immunologie, Faculté de médecine, Université Laval, Centre de recherche du CHUQ, 2705 Laurier blvd., Québec City (Québec), G1V 4G2, Canada; E-Mail: Luc.Bissonnette@crchul.ulaval.ca; 2 Centre de recherche en infectiologie de l'Université Laval, Centre de recherche du CHUQ, 2705 Laurier blvd., Québec City (Québec), G1V 4G2, Canada

**Keywords:** infectious diseases, molecular diagnostics, point-of-care, personalized medicine

## Abstract

Infectious disease management essentially consists in identifying the microbial cause(s) of an infection, initiating if necessary antimicrobial therapy against microbes, and controlling host reactions to infection. In clinical microbiology, the turnaround time of the diagnostic cycle (>24 hours) often leads to unnecessary suffering and deaths; approaches to relieve this burden include rapid diagnostic procedures and more efficient transmission or interpretation of molecular microbiology results. Although rapid nucleic acid-based diagnostic testing has demonstrated that it can impact on the transmission of hospital-acquired infections, we believe that such life-saving procedures should be performed closer to the patient, in dedicated 24/7 laboratories of healthcare institutions, or ideally at point of care. While personalized medicine generally aims at interrogating the genomic information of a patient, drug metabolism polymorphisms, for example, to guide drug choice and dosage, personalized medicine concepts are applicable in infectious diseases for the (rapid) identification of a disease-causing microbe and determination of its antimicrobial resistance profile, to guide an appropriate antimicrobial treatment for the proper management of the patient. The implementation of point-of-care testing for infectious diseases will require acceptance by medical authorities, new technological and communication platforms, as well as reimbursement practices such that time- and life-saving procedures become available to the largest number of patients.

## 1. Introduction

Despite significant advances in sanitation and medicine, infectious diseases still annually claim in excess of 15 million lives. In the 20th century, antimicrobial therapy has provided medicine with powerful tools to combat infectious diseases, but the promises of antimicrobial agents have been hampered by the development of resistance and more recently, of multiple resistance to widely used drugs, induced by an overutilization of broad-spectrum antibiotics and complicated by the drought in the antimicrobial drug pipeline [1,2,3,4,5,6]. Today, as in the times of Louis Pasteur, the identification of a microbe causing an infection may still require 2–3 days since the penetrance of rapid molecular diagnostics has been limited by a number of factors, such as cost and cultural resistance. This lack of speed in clinical microbiology has led to empirical therapy practices to which the emergence of super-resistant microbes can be attributed for the most part. Other confounding factors contributing to the evolution and dissemination of emerging and multiresistant pathogens on the global scale have been identified by Morens *et al.* [7].

## 2. Personalized Medicine for Infectious Diseases?

Personalized medicine is generally described as a discipline that relies on the genomic information of an individual to guide the prescription of an appropriate therapeutic regimen in perspective with her/his anticipated response to a particular drug or combination of drugs *i.e.*, to provide the right drug, at the right dosage, to the right patient. Traditionally, personalized medicine concepts and strategies have focused on the management of genetic diseases or chronic disorders where polymorphisms in genes controlling Phase 1 and/or Phase 2 drug metabolism are interpreted rationally against growing databases of known pharmacological interactions between drugs and proteins with altered function(s), to guide drug prescription and dosage [8,9,10,11]. Increasing knowledge in pharmacogenetics is used to develop companion diagnostics based on the determination of other clinically-relevant biomarkers, oncogenes or viral receptors for examples.

Infectious diseases are rarely considered as model applications of personalized medicine, as evidenced in the document *The Case for Personalized Medicine* [11] in which very few of listed theranostic tests address infectious diseases. However, this perception is slowly changing as the utility of biomarkers linked to the immune response, infectious disease susceptibility, host-microbiota interactions, or hypersensitivity to antimicrobial drug treatment is being demonstrated [10,12]. Personalized medicine for infectious diseases possesses obvious advantages to orient the molecular management of infections. Indeed, the application of a personalized medicine approach could be envisioned as a bimodal process aiming at deciphering clinically-relevant genomic components of the disease-associated pathogen(s) and of the patient, to select and optimize the course of treatment of acute life-threatening diseases. First and foremost, molecular microbiology offers technologies enabling the rapid detection and/or identification of microorganisms including fastidious and unculturable pathogens, crucial information that a physician can readily exploit to orient the first (critical) hours of a patient's therapeutic regimen and significantly accelerate the management of the infection [4,13,14,15]. Upon phenotypic or genotypic determination of the drug resistance or toxin production potential by other (conventional) means, therapeutic option(s) could be re-evaluated but, for a large number of cases, the time-effectiveness of the molecular microbiology intervention will have already increased the chances of survival of the patient. The accurate determination of antimicrobial susceptibility patterns will still rely on phenotypic methods but, while the genotypic determination of the antimicrobial resistance potential of Gram-positive pathogens (e.g., methicillin or vancomycin) may offer more opportunity for yielding fast results, the strategy applicable to Gram-negative bacteria is complicated by the higher variety of resistance mechanisms and the genetic drift of resistance gene alleles that limit the spectrum of antibiotic options. In parallel, the determination of the pharmacogenetic profile of the patient may provide an additional assessment of the drug metabolizer phenotype and/or of the risk of potential adverse drug interactions [1], while the determination of the immunogenetic profile of the patient could be used to evaluate her/his susceptibility to infection [16,17]. 

A good example of an integrated personalized medicine strategy addresses the rapid detection of *Streptococcus agalactiae* in parturient women with the BD GeneOhm™ StrepB test where a positive test provides the physician with the indication to initiate an appropriate antibiotic regimen prior to baby delivery in order to prevent neonatal infections [18]. In the clinical microbiology market, this real-time polymerase chain reaction (rtPCR) assay was followed by several other BD GeneOhm™ tests, initially developed by our group, for the rapid diagnostics of hospital-acquired infections associated to methicillin-resistant *Staphylococcus aureus* (MRSA), vancomycin-resistant enterococci (VRE), and *Clostridium difficile*. It has been shown that these tests have saved lives, decreased the spread of these infections, and reduced healthcare costs [19,20,21,22,23,24,25,26].

Although not identified as such, personalized medicine has also been driving the management of HIV/AIDS since 2001, in the form of the TruGene™ (Siemens Healthcare Diagnostics) or ViroSeq™ (Abbott Molecular) nucleotide sequencing tests that are used to adjust the antiviral treatment of HIV-seropositive and AIDS patients according to the genotype of the virus circulating at the time of testing, upon interrogation of a database of known antiviral drug resistance mutations [27]. 

There is also evidence suggesting that the management of tuberculosis could be dictated by an integrated personalized medicine approach taking into account genetic information from both the microbe and the infected individual, to better exploit the potential of molecular diagnostics. For example, while the recently introduced Xpert^®^ MTB/RIF test (Cepheid) can provide rapid *Mycobacterium tuberculosis* identification and primary assessment of the drug multiresistance profile [28,29], it has been suggested that the *N*-acetyltransferase 2 genotype of the patient may be used to determine her/his pharmacogenetic profile, to guide the isoniazid dosage, and limit drug hepatotoxicity [11,30,31,32]. Finally, the genotyping of several immunogenetic targets could provide additional information on human susceptibility to infection and disease severity [33]. 

## 3. Infectious Disease Management in the Molecular Medicine Era—Shortening the Diagnostic Cycle

**Figure 1:**
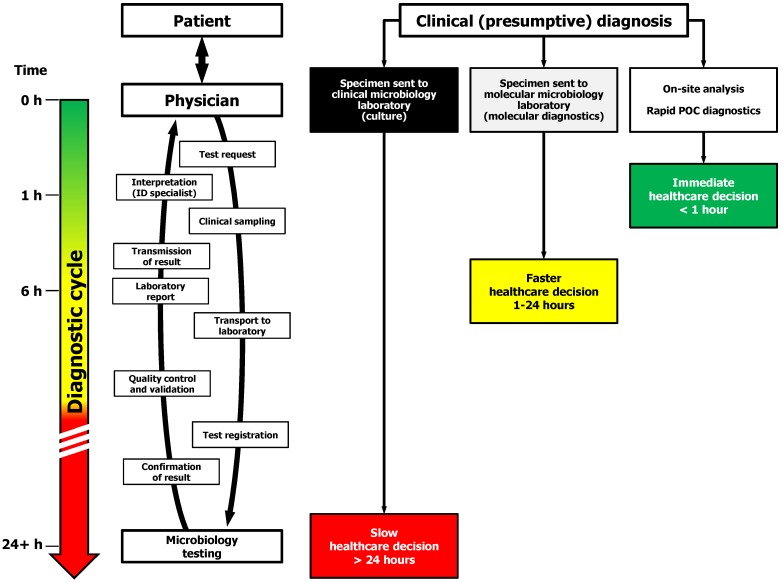
Personalized medicine in infectious disease management. The implementation of rapid point-of-care (POC) microbiology shall decrease the length of the diagnostic cycle in order to accelerate infectious disease management.

In the healthcare system, the arrival of a febrile, potentially infected, patient initiates a diagnostic cycle consisting of several time-consuming steps (see Figure 1). While with classical phenotypic microbiology identification methods, it is accepted that the time to perform the analytical phase of the cycle is the most important temporal limitation, there are also important delays associated with the pre- and post-analytical phases such as sample transport, batching practices, and result transmission which inherently augment the turnaround time [34,35]. 

One of the rationales for implementing rapid molecular microbiology in clinical settings is to compensate for the extended period of time, *i.e.*, at least 24 hours that is required by culture-based microbiology to deliver a putative microbe identification, such as a positive blood culture Gram stain result which may be sufficient to initiate an empirical therapy before identification confirmation and phenotypic information about antimicrobial susceptibility are obtained. In many instances, experience-based empiric management gives good results, but on the downside, if the choice of antibiotherapy is inappropriate or the treatment is initiated too late, the outcome is often treatment failure [3,4,36,37]. The empiric management of infections is a clinical practice recognized to have induced an overuse of broad-spectrum antibiotics that (1) increased the selective pressure on microorganisms which in return evolved and/or transmitted antimicrobial resistance mechanisms and genes that threaten the efficiency of the last weapons available to combat "superbugs" such as vancomycin, (2) contributed to the emergence of drug-resistant hospital-acquired infections which unnecessarily claim hundreds of thousands of lives annually, and (3) are responsible for severe complications like allergy or disturbance of the normal microbiota. For example, it was shown that 89% of patients with laboratory-confirmed flu diagnostics had received unnecessary (and not effective) antibacterial therapy upon admission in a Canadian hospital [38]. 

In the daily practice of medicine, if the result of a microbial identification test performed on a very symptomatic patient would be available within an hour or two, the physician would dispose of highly useful information to initiate an appropriate therapy. In addition to speed, another major advantage of molecular microbiology over classical microbiology lies in its capacity to detect fastidious or unculturable microorganisms [15], and to determine if the patient is fighting a polymicrobial infection [39,40,41]. 

In this context, the main cultural challenge to overcome is the time required to complete the diagnostic cycle (Figure 1). Shortening the diagnostic cycle to less than 2 hours, ideally between 30 to 60 minutes, could be realized through the implementation of more rapid (molecular) diagnostic tests performed closer to the patient, ideally at bedside. It is anticipated that point-of-care (POC) infectious diseases diagnostics will be eventually feasible using simple sample-to-answer instruments incorporating the most critical steps of a molecular diagnostic method, to provide specific and sensitive answers with minimal delays [42]. Awaiting these revolutionary miniaturized personalized healthcare tools, we believe that near POC laboratories should be equipped and certified to offer a comprehensive menu of commercially-available molecular diagnostics tests in hospital departments where the impact and cost-effectiveness will be greater, or in (private) medical clinics. A proof of concept of this operational change in culture is being realized in Marseille, France [43,44]. 

Nucleic acid-based tests and molecular microbiology methods amenable to near POC for infectious disease management come in different methodological configurations requiring relatively high technical skills which may impose a higher level of procedural and physical confinement. Real-time PCR was the first technology approved for clinical microbiology testing that enabled microbial detection and/or identification in less than 1 hour directly from a clinical sample [18,45]. With the number of tests currently approved by the U.S. Food and Drug Administration (FDA) for *in vitro* diagnostics [46], this technology should be offered at near POC to demonstrate its potential impact on disease management. In the case of the GeneXpert™ technology, the reduction in the number of technical steps required for performing rtPCR in an integrated cartridge was certainly critical to the FDA approval and to the moderate complexity status (under CLIA) of the instrument, thereby opening a breach in conventional clinical microbiology testing for more complex but less labor-intensive sample-to-answer diagnostic systems such as the BD MAX™ System of BD Diagnostics GeneOhm [47]. On the other hand, the clinical utility of the xTAG^®^ tridimensional array multiparametric detection platform of Luminex Molecular Diagnostics [48] was probably raised as a critical element for its regulatory approval by the FDA, despite the methodological complexity of performing an independent multiplex PCR amplification before molecular hybridization [49,50,51,52]. 

More recently, two other technologies, less compact but possibly implantable in near POC laboratories have emerged in infectious diseases molecular diagnostics: 1° mass spectrometry capable of delivering post-blood culture microbial identification in a matter of minutes [53,54,55,56], and 2° next-generation sequencing that demonstrated its usefulness during the 2011 Germany *Escherichia coli* O104 outbreak associated to contaminated foodstuff [57]. Despite their power of analysis, matrix-assisted laser desorption/ionization time-of-flight (MALDI-TOF) mass spectrometry and next-generation sequencing must be performed mainly on cultures, thus delaying the diagnostic cycle by at least 18–24 hours. However, microbial identification directly from clinical sample has been demonstrated by the electrospray ionization mass spectrometry of broad range PCR amplicons (PCR-ESI) [53,58].

Theoretically, a reduction in the number of human interventions and physical proximity shall accelerate the availability of results, thereby leading to shorter turnaround time and more effective infectious disease management. The cost-effectiveness of near POC diagnostics will require further health economics studies, but when one takes into consideration that the cost of a complicated sepsis case can exceed 500,000 USD [59], we sincerely believe that investing in qualified staff, laboratory upgrade, and multiparametric detection technologies are worth it, especially for the management of life-threatening infections, neonatal infections, and those of immunocompromised individuals, especially in the context that many infections might be polymicrobial [39,40,41]. Healthcare organizations that are not ready to invest in diagnostic technologies enabling microbial detection directly from clinical samples should possibly consider investing in MALDI-TOF since rapid and accurate microbial identification from primary culture may also contribute to an accelerated and cost-effective management of infected patients. 

## 4. Molecular Tools for POC or near POC Diagnostics of Infectious Diseases

Point-of-care testing can be defined as *patient specimens assayed at or near the patient with the assumption that test results will be available instantly or in a very short timeframe to assist caregivers with immediate diagnosis and/or clinical intervention* [60]. This definition clearly indicates that distance and time are essential features onto which technology experts and healthcare system authorities should focus to shorten the diagnostic cycle and make molecular POC testing a reality. Bedside testing might constitute the ultimate goal, but the development of near POC laboratories would certainly shorten the diagnostic cycle and increase the efficacy of infectious diseases management by improving access to highly efficient nucleic acid-based tests.

The current market for infectious diseases POC testing is dominated by rapid microscopy or immunological diagnostic tests that can be realized outside clinical laboratories, but often lack in sensitivity and/or specificity [61,62,63,64,65,66,67]; a regularly updated list of Clinical Laboratory Improvement Amendments (CLIA)-waived tests can be downloaded from the Internet [68]. Procalcitonin, a promising biomarker that is used in clinical practice in some countries, provides indications of the presence and severity of bacterial infections such as community-acquired pneumonia and sepsis [69,70]. Although not specific and despite some contradictory reports regarding its accuracy and usefulness as a sepsis diagnostic marker, the suggestion that procalcitonin serum levels could be used as an antimicrobial stewardship tool has been made [69,70]. So far, not a single nucleic acid-based true POC test has reached the (CLIA-waived) clinical microbiology market [64,68,71] although major efforts are undertaken to circumvent the formidable challenge of developing user-friendly platforms capable of detecting microbial pathogens present in low concentrations in putatively infected samples... with the simplicity of a pregnancy or personal glucose test.

In the last ten years, many nucleic acid-based tests have been approved for clinical diagnostics by the FDA [46] after the demonstration of their equivalence to and/or superiority over gold standard methods, with respect to analytical performance (sensitivity and specificity) and predictive values; speed not always being a decisive factor in determining the clinical utility of a test. The preferred platform is rtPCR, a technology operating in a closed vessel format that virtually eliminates cross-contamination of laboratory space by amplification products, but limited by the number of targets it can simultaneously detect. In the current philosophy (or art) of infectious disease management, rtPCR provides a very good platform for the detection of a pathogen in less than two hours, if it is specifically suspected by the treating physician. Under CLIA regulations, instruments for performing nucleic acid-based tests have been given either moderate- or high-complexity status, thus precluding their use at POC, since the nature and number of technical steps, and system maintenance and troubleshooting require more qualified staff [67]. To alleviate some of the instrumental burden of thermal cycling, significant advances in isothermal amplification procedures have been demonstrated [67], but to achieve efficient detection, sample preparation must be done externally or at the same temperature thereby complicating this important procedural step.

Notwithstanding the fact that molecular diagnostic assays must be analytically and clinically equivalent or superior to gold standard procedures, the implementation of true molecular POC testing for infectious diseases will necessitate a major change in culture such that diagnostic interpretation, therapeutic management decision(s), and antimicrobial treatment (prescription) could be delegated to medical staff other than microbiologists and clinicians. However, envisaging near POC testing in decentralized laboratories of hospitals (intensive care units, pediatrics department, *etc.*) or parallel healthcare (medical clinics, pharmacies, nursing homes, *etc.*) is an approach which could ensure that diagnostic results be returned to the test requestor as quickly as possible, ideally within 1–2 hours, *i.e.* ,before a potentially late or wrong clinical management decision would have been taken [34,43,44,62,72,73]. 

In light of the (FDA) regulatory approval constraints, most *in vitro* diagnostic tests target a limited number of microbes. However, it must be highlighted that several syndromic infections such as bloodstream, respiratory, or urinary tract infections are potentially caused by a large spectrum of viruses, bacteria, fungi or parasites, and seldom polymicrobial [39,40,41]. The management of these infections can be accelerated by multiparametric detection platforms, such as xTAG^®^ of Luminex Molecular Diagnostics [48] or the eSensor^®^ respiratory viral panel of GenMark Diagnostics [74] that can interrogate a sample for the presence of a disease-causing microorganism known to be part of a syndrome-associated microbial panel, instead of performing multiple tests which would increase the cost. Microarray hybridization is considered to be a cost-effective platform with a good probability of success in multiparametric detection. Performing this type of bioanalysis implies that nucleic acids extracted or purified from microbial targets must be amplified, and perhaps, labeled externally before amplification products are hybridized to a bidimensional (microchip) or tridimensional (beads) array of capture probes. Array scanning or imaging is used to decipher the microbial content of the sample [50,53,75,76,77]. 

Multiparametric detection platforms can accomplish what they are intended for as long as extremely important issues dealing with the preparation of microbial nucleic acids from human clinical samples and molecular contamination of the sample or of the testing environment are taken into account:

(1) sample preparation shall enable the concentration of microbes and/or the recovery of intracellular pathogens. Indeed, achieving this should prevent the detection of (soluble) DNA liberated by dead or damaged pathogens exposed to antibiotics and improve the probability of detection of a microbial target against a lesser background of human DNA. The significance of microbial DNAemia is seldom raised against the utilization of PCR in clinical microbiology but, for the management of life-threatening infections, we concur with Bauer and Reinhart [78] in the sense that *"... presence of a pathogen-associated DNA amplicon is a meaningful event in severe sepsis and warrants further investigation as to its suitability to guide anti-infective therapy"*. Alternatively, DNA from dead cells could be inactivated by compounds such as EMA or PMA (ethidium or propidium monoazide) [79]. However, EMA can penetrate the membrane of viable cells, EMA uptake is species-dependent, and there are drawbacks with PMA utilization [80];
(2) the recovery of pathogens and nucleic acid extraction from a relatively large sample volume, for example 1 to 30 mL in the case of neonatal or human bloodstream infections is a major challenge that might require some external sample pretreatment, in order to deliver a concentrated subsample containing the target analyte(s) more easily subjected to the amplification and detection processes;
(3) nucleic acid extraction or purification must enable the removal of PCR inhibitors known to hinder the performance of enzymatic components, and;
(4) strict precautions to control the cross-contamination of personnel and equipment by amplification products that would negatively affect the performance and clinical validity of the test.

The introduction of multiparametric detection platforms in *in vitro* diagnostics shall pave the way to *stat* molecular diagnostic tests performed by minimally-trained medical staff or dedicated technical staff with user-friendly compact automatic diagnostic systems operated at near POC, as soon as a sample is submitted for analysis [16,34,43,44,81,82,83]. Conceptually, these sample-to-answer automated systems shall be designed to accept a biologically significant clinical sample, proceed to the extraction/purification of specific analytes, and perform a certain number of bioanalytical steps, e.g., PCR amplification and/or microarray hybridization, to reveal the presence and/or determine the concentration of a specific microbial (genomic) target. In the clinical microbiology market, examples of automated fluidic systems accommodating a large number of clinical *in vitro* diagnostics tests comprising sample preparation and molecular amplification by rtPCR include the GeneXpert™ system of Cepheid [28] and the recently approved BD MAX™ of BD Diagnostics GeneOhm [16,47,67,81,84]. While the former system has been developed for near POC applications by proposing tests from relatively simple samples (swabs or swab contents in elution buffer), the latter platform offers more flexibility due to its capacity for the extraction and purification of nucleic acids from more complex biological samples. However, both systems are limited by the volume of crude sample that can be handled efficiently, thereby imposing some sample pre-treatment (microbial concentration, removal of human cells, *etc.*) before loading in the instrument.

Microfluidics is a relatively adaptable technology that has provided analytical (bio)chemistry with miniaturized devices capable of streamlining analytical processes. To accede to the near POC status, we believe that the next generation of diagnostic instruments will drive sample-to-answer devices bearing modules enabling sample pre-treatment, preparation, nucleic acid extraction, and microarray hybridization with or without molecular amplification of microbial genomic targets. The requirements for future miniaturized lab-on-a-chip platforms operating at near POC impose the integration of many technical steps typically performed in clinical laboratories, such that a diagnostic result is provided hopefully within 1 hour after sampling [45,82,86,87,88,89,90,91,92]. This task will not be trivial, as there are numerous ways to combine microfluidic components and strategies to address the requirements for near POC testing: 1° rapid prototyping and (mass) microfabrication [92,93], 2° microfluidic circuitry, pumping, and valving [94,95,96,97], 3° sample preparation and cellular lysis schemes leading to extraction, concentration and/or purification of nucleic acids [98,99,100,101,102,103], and 4° molecular (isothermal) amplification and/or molecular hybridization [104,105,106,107,108,109]. Especially for the management of infectious syndromes at near POC, technology developers must deal with the challenging engineering tasks of (1) handling biochemically complex samples with relatively large volume (1–30 mL), (2) reducing the sample volume by analyte concentration or purification, and (3) integrating mechanical, thermal, and optical detection processes, to ensure an adequate analytical sensitivity, clinical validity, and user-friendliness of the test. In addition, research efforts should also be devoted to the development of world-to-chip interfaces and automated result reporting, interpretation, and data transmission such that the diagnostic cycle is closed as promptly as possible.

In the last several years, our research group and GenePOC, a young start-up company, have been developing integrated microfluidic centripetal device (MCD) technology platforms that are designed to encompass most of the abovementioned requirements and to operate at POC. The versatility of the MCD technology shall make it applicable not only to nucleic acid-based tests, but also to protein biomarker-based methods [45,86,89].

## 5. Applications and Anticipated Impact of POC or near POC Diagnostics of Infectious Diseases

A greater penetration of rapid molecular diagnostics of infectious diseases in the healthcare system of developed and developing countries offers the promises of faster disease management, more adequate antimicrobial therapy, better allocation of healthcare human and laboratory resources, and less morbidity, mortality, and costs. Depending on the type of healthcare system, the (administrative) compartmentalization of healthcare facilities budget practices constitutes a major obstacle to the implementation of rapid molecular diagnostics in the sense that assay costs are taken into account without considering the mid-to-long term impacts of the technological advance on the health of the clientele and the efficiency of the organization. In this era of exploding healthcare costs, the arrival of novel technologies and methodological practices cannot be done without thoughtful planning, such that the rational choices initially made will serve to demonstrate the cost-effectiveness and clinical usefulness and motivate further development within the organization of clinical microbiology and infectious disease care. This section comprises examples of clinically-relevant applications of POC or near POC molecular diagnostics which may serve to benchmark the personalized medicine of infectious diseases.

### 5.1. Hospital-Acquired Infections

In recent years, hospital-acquired (nosocomial) infections have become a major concern in healthcare facilities worldwide, their management being greatly complicated by the emergence of resistant and multiresistant Gram-positive (methicillin-resistant *S. aureus*, vancomycin-resistant enterococci, and *C. difficile*) and Gram-negative (*E. coli*, *Klebsiella pneumoniae*, *Enterobacter* spp., *Serratia marcescens*, *Pseudomonas aeruginosa*, and *Acinetobacter baumanii*) pathogens [4]. In the United States in Year 2000, it was estimated that 1.7 million individuals acquired an infection in a hospital and approximately 100,000 died from it, translating into at least $6.5 billion in healthcare expenditures [110].

In the case of Gram-negative hospital-acquired pneumonia, Arnold *et al.* [1] recently addressed the timeliness of antimicrobial empiric therapy and the necessity of limiting the likelihood of adverse events and drug interactions. Considering the fact that nosocomial pneumonia constitutes the second most common infection among hospitalized patients in the United States and that inappropriate initial antimicrobial therapy has been associated with decreased survival of patients [111], the application of rapid multiparametric molecular diagnostics could be highly significant [51].

### 5.2. Bloodstream Infections and Sepsis

Bloodstream infections are life-threatening situations for which the critical time window for appropriate management is estimated to be less than 6 hours. Indeed, it has been demonstrated that every hour gained to initiate an appropriate antimicrobial therapy of febrile patients significantly increases the probability of survival [36,112]. Blood culture, the gold standard method, has a very high positive predictive value but, in light of the load and culturability state of bacterial and/or fungal pathogens, the overall positivity rate for the diagnosis of bloodstream infections is estimated to only 30–40% [13] and perhaps as low as 20% [78]. In a recent study, Brown and Paladino [113] have reviewed the literature pertaining to management of MRSA bacteremia in the United States and European Union, especially in the context where PCR is used to guide treatment. The main conclusions of this study are that PCR has the potential to reduce MRSA-induced mortality rate, while being less costly than the empirical therapy approach. In another comparative study conducted at the Ohio State University Medical Center, Bauer *et al.* [114] have quantified the impact of rtPCR used to confirm the presence of MRSA in positive blood cultures: length of stay of patients diagnosed with MRSA bacteremia was 6.2 days shorter and the mean hospital costs were $21,387 less per case. The cost-effectiveness and positive impact on mortality rate resulting from the management of sepsis by rapid molecular diagnostics is also supported by a mathematical prediction model [14].

Strategically, performing the detection of MRSA on positive blood cultures is faster than current culture-based procedures, but the timeliness of PCR-based detection of MRSA could be even more important if detection was achieved directly from blood. In a recent review, three commercially-available molecular amplification tests theoretically capable of detecting bloodstream pathogens in less than 12 hours have been compared, SeptiTest, Septi*Fast*, and VYOO/LOOXSTER [115]. As the authors have pointed out, with respect to procedural elements which lengthen the diagnostic cycle, the potential of these tests to accelerate the management of bloodstream infections and better guide antimicrobial therapy is diminished by batching procedures which extend the turnaround time to 18 hours at best. 

### 5.3. Influenza and Severe Respiratory Tract Infections

The management of influenza is a recurrent annual problem in the healthcare system, as clinical symptoms evaluation seldom leads to unnecessary and ineffective antibacterial therapy [38]. In the perspective that antiviral treatment is more effective when initiated within 48 hours after symptom onset and that nucleic acid-based tests (reverse transcription PCR) are more rapid than culture and more sensitive than commercial antigen-based assays, it would be rational to advocate for a non-empirical strategy providing the larger benefits. Indeed, while influenza molecular diagnostics may provide very timely results and lead to reduced antibiotic use and hospital admissions, an empirical antiviral therapy strategy, costing approximately the same as RT-PCR, would result in the treatment of 5–15 patients without influenza for each positive case [116]. Other viral respiratory tract infections caused by at least 15 different viruses are now being diagnosed through molecular testing and, in a recent report, the Infectious Diseases Society of America expressed the need for more rapid molecular tests in this clinical field [117].

### 5.4. Other Clinical Indications and Strategic Suggestions for POC or near POC Testing Implementation

To ensure a proper utilization, sustainability, and better penetrance of molecular diagnostics at POC or near POC, we believe that decentralized laboratories should offer a highly strategic choice of technologies and tests, while we await the arrival of real POC molecular testing. Here are suggestions of infrastructures minimally anticipated in healthcare institutions. First, obstetrics wards should offer 24/7 rapid rtPCR tests (BD GeneOhm™ StrepB or Xpert^®^ GBS) for the detection of *S. agalactiae* (Group B streptococcus) in parturient women, as it has been shown that GBS detection 2–3 weeks before delivery has a sensitivity of approximately 50% due to the fact that GBS carrier status of women change (negative to positive and the inverse) in the interval. Second, intensive care units and pediatrics departments should incorporate in their patient management plan a 24/7 access to state-of-the-art molecular microbiology for performing multiparametric rtPCR and/or molecular hybridization, directly from clinical samples, for the rapid diagnosis of bloodstream (neonatal) infections and complicated respiratory infections. If a near POC molecular microbiology laboratory is not considered an option, post-blood culture microbial identification by MALDI-TOF should be performed in the clinical microbiology laboratory. Alternatively, and this is more controversial, the universal screening of MRSA, VRE, and perhaps *C. difficile* should be ideally done upon admittance into a mid-to-long duration care department, to prevent the dissemination of healthcare-acquired infections. The emergency room would be an ideal site for the rapid POC screening of hospital-acquired infection pathogens, upper and lower respiratory tract infections, sexually transmitted diseases, urogenital infections, and diarrhea, as it would accelerate the flow of patients and reduce long waiting hours. Ultimately, simple POC devices and tests will not only be used in hospitals, but in large medical clinics, pharmacies, and in remote areas (developed countries).

## 6. Regulatory, Ethical, and Financial Challenges to POC or near POC Testing for Infectious Diseases

In the last ten years, molecular diagnostics has proven to be a transforming discipline in clinical microbiology, catalyzed by the regulatory acceptance of rtPCR as the driving technology capable of accelerating the management of infectious diseases. In fact, molecular diagnostics is a critical component of the emerging concept of "precision medicine" [118,119] and, as in other fields of medicine, molecular diagnostic technologies and tests are not expected to replace all conventional microbiology procedures. For extreme medical situations like septicemia however, molecular diagnostics shall provide complementary tests that will improve patient management and save lives. 

In terms of regulatory approval, we believe that the approval of the Xpert^®^ tests and instruments by the U.S. Food and Drug Administration and the moderate-complexity status given under CLIA '88 regulations constitute major milestones that will have paved the way to other microfluidic systems and devices designed for performing molecular microbiology at near POC, especially if equivalence to or superiority over gold standard method(s) is demonstrated [73,120,121,122]. Awaiting the ultimate CLIA-waived personalized medicine specific rtPCR-based microfluidic devices which could be operated at POC by anyone, even the patient, technology developers shall elaborate very strict requirements to enable the approval of multiparametric devices bearing diagnostic microarrays having the potential to perform more tests per unit of time and augment the probability of detection of a sepsis-associated microbe, for example. For the time being, technology developers should target a moderate-complexity status which may enhance the penetration of their instruments and tests in the decentralized healthcare market, since nucleic acid-based testing requires minimally trained staff for operation [71]. 

The acceptance of newer technologies cannot be done without a proper evaluation of the costs. Clinical microbiology has the opportunity to literally "renovate" itself and this does not only imply the purchase of expensive instruments, the upgrade of laboratories in response to the confinement requirements of molecular microbiology, and the daily operation of these infrastructures, but also an intellectual "price" imposed by the training of staff. POC devices and tests shall alleviate many of the abovementioned obstacles.

Inasmuch as the personalized medicine management of infectious diseases principally targets the genomic components of the pathogen(s), the determination of the pharmacogenetic and/or immunogenetic profiles of a patient might provide the physician with additional clues to adequately fight an infection, we do not believe that infectious diseases personalized medicine will be hampered by the social issues and ethical debate that eventually led to the approval of Genetic Information Nondiscrimination Act (GINA) of 2008 by the United States legislative bodies [123,124,125].

## 7. Conclusions

In this article, we have presented a personalized medicine model by which patients could greatly benefit from improved infectious diseases management practices guided by clinically-relevant genomic information extracted from microbes in specialized POC devices and tests done near patients, or in near POC laboratories and rapidly relayed to the treating physician, to alleviate time-consuming and error-prone interventions occurring in the pre- and post-analytical phases of clinical microbiology testing. 

In this context and in the expectation of real CLIA-waived POC testing, we suggest that healthcare systems initiate near POC testing pilot programs proposing a menu of nucleic acid-based tests approved by regulatory authorities, to demonstrate the advantages of this "culture without culture" changing concept. In addition, feasibility studies should be supported by pharmacoeconomic studies for 1° demonstrating the socio-economical potential of the approach to decrease the unnecessary morbidity and mortality associated with life-threatening infections for which the empiric management and inappropriate antimicrobial therapy often lead to treatment failure with dramatic consequences, and 2° accumulating evidence for convincing governing bodies and insurance companies to reimburse testing costs [126]. 

Finally, and according to the principles of POC testing, we also believe that near POC laboratories could also provide a research and validation platform for (showcasing) the next generation of simple-to-operate, yet technologically sophisticated, compact microfluidic systems *en route* to the ultimate goal of true point-of-care molecular medicine.
